# ﻿Two new species of *Landouria* Godwin-Austen, 1918 (Gastropoda, Camaenidae) from Thailand, with a key to Thai species

**DOI:** 10.3897/zookeys.1208.117056

**Published:** 2024-08-05

**Authors:** Benchawan Nahok, Utain Chanlabut, Sakboworn Tumpeesuwan, Chanidaporn Tumpeesuwan

**Affiliations:** 1 Department of General Science, Faculty of Education, Chaiyaphum Rajabhat University, Muang District, Chaiyaphum, 36000, Thailand Chaiyaphum Rajabhat University Chaiyaphum Thailand; 2 Department of Biology, Faculty of Science, Mahasarakham University, Kantharawichai District, Maha Sarakham, 44150, Thailand Mahasarakham University Maha Sarakham Thailand

**Keywords:** Aegistini, Bradybaeninae, radula, reproductive anatomy, shell morphology

## Abstract

The present work contains descriptions of two new species of *Landouria* and a key to Thai species of this genus. *Landouriabella***sp. nov.** is described from isolated limestone hills in Ratchaburi and Phetchaburi provinces, western Thailand. This new species is characterized by its small depressed-globose shell without a peripheral keel, the presence of a small, thin lamella on the columellar side of the inner aperture, a long, cylindrical distally bent flagellum, a short penis with a rounded verge, and a short, thick free oviduct and vagina. The second new species, *Landouriamonodon***sp. nov.** is described from sandstone hills in Kalasin Province, northeastern Thailand. This species has a depressed-conic shell with a blunt peripheral keel, a thick prominent lamella on the columellar side of the inner aperture, a short, finger-shaped, distally bent flagellum, and a long penis with a small, short verge.

## ﻿Introduction

*Landouria* Godwin-Austen, 1918 is a terrestrial snail genus that is widely distributed in mainland East and Southeast Asia and the Indo-Australian Archipelago (Schileyko 2004; Hirano et al. 2014; Nurinsiyah et al. 2019). The genus is conchologically characterised by having a depressed, usually brownish-corneous shell, with often angular or keeled whorls, a wide umbilicus, and 5–6 moderately convex whorls. The last whorl is usually somewhat deflected and evenly rounded to obtusely angulated at the periphery. The genus is anatomically characterised by the absence of a dart sac and accessory organs, but also by having a short penis, epiphallus, flagellum, and vas deferens; the gametolytic sac is swollen at the base; there is a thin duct at the middle and a globose sac at the distal end (Godwin-Austen 1918).

Seven species of *Landouria* have been previously reported in Thailand as Aegista (Plectotropis) spp. (Solem 1966; Panha 1996; Hemmen and Hemmen 2001) (see Tumpeesuwan and Tumpeesuwan 2019: table 2). Subsequently, one species has been placed in the genus *Thaitropis* Schileyko, 2004, as *Thaitropisgoniochila* (L. Pfeiffer, 1862), based mainly on the presence of the sharp constriction between the epiphallus and the penis. However, Nurinsiyah et al. (2019) synonymized *Thaitropis* with *Landouria*. Nahok et al. (2021) suggested that *Thaitropis* sp. (in Sutcharit et al. 2017: fig. 6–32e) should be classified as *Landouriadiplogramma* (Möllendorff, 1902), based on a comparison with voucher specimens deposited in the Naturalis Biodiversity Center (Leiden, the Netherlands), which is labelled as *Plectotropisdiplogramme* Möllendorff, 1902 (RMNH.MOL.309867). Preserved specimens from Khao See Siad Ah, Nakhon Ratchasima (NHMSU-00034) (Nahok et al. 2021: figs 3F, 4F) exhibiting shell characters closely correspond with these voucher specimens of *P.diplogramme*. These preserved specimens have a reproductive anatomy that is consistent with typical features of *Landouria*. Therefore, these specimens should be identified as *Landouriadiplogramma*.

Boonngam et al. (2008) reported *L.winteriana* (L. Pfeiffer, 1842) and *L.smimensis* (Mousson, 1849) in eastern Thailand. However, both species were previously reported from Sumatra (van Benthem Jutting 1950, 1959; Zilch 1966; von Martens 1867; Schileyko and Kuznetsov 1998; Heryanto 2013; Marwoto 2016; Köhler et al. 2019; Nurinsiyah et al. 2019). Köhler et al. (2019) hypothesized that *L.winteriana* (in Boonngam et al. 2008) may have been confused with an undescribed species, which indicates that there may be additional cryptic species in other localities of *L.* “*winteriana*”. Nurinsiyah et al. (2019) concluded that *L.smimensis* is found only in the Tengger Mountains of East Java, whereas the other records in Central and East Java, Lombok, Bali, and Thailand (Rensch 1931, 1934; Vermeulen and Whitten 1998; Boonngam et al. 2008) most likely represent separate species. Therefore, a comprehensive re-assessment of *L.* “*winteriana*” and *L.* “*smimensis*” in Thailand is required, and it is necessary to re-examine both species as reported by Boonngam et al. (2008).

Recently, six *Landouria* species have been described from northeastern Thailand based on the characters of shell, radula morphology, and reproductive anatomy. These are *L.strobiloides* Tumpeesuwan & Tumpeesuwan, 2019 and five species described by Nahok et al. (2021): *L.circinata*, *L.tuberculata*, *L.trochomorphoides*, *L.chloritoides*, and *L.elegans*.

**Figure 1.**
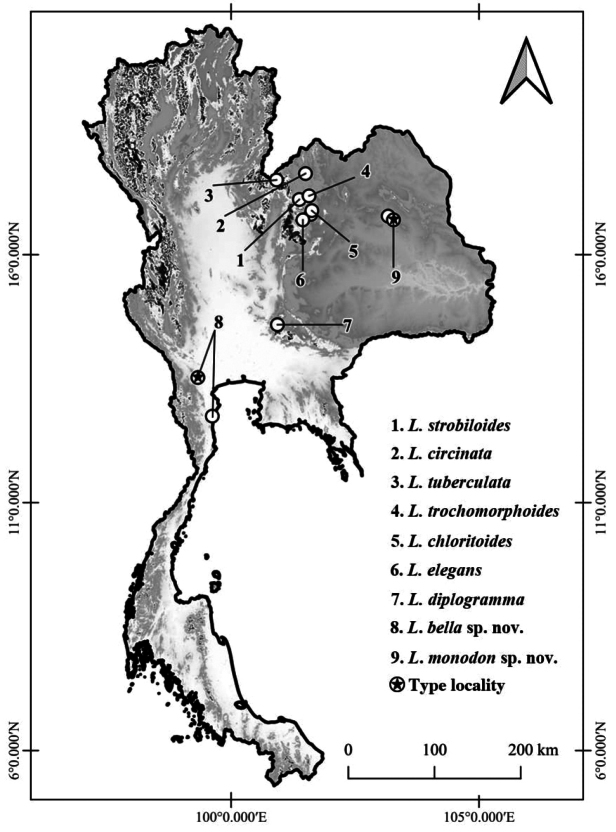
Geographic distribution of verified Thai *Landouria* species and showing the type localities of *L.bella* sp. nov. and *L.monodon* sp. nov.

In this study, we describe two new species, which were discovered on limestone hills in western Thailand and sandstone hills in northeastern Thailand (Fig. 1).

## ﻿Materials and methods

This study is based on materials collected during surveys by Nahok (2020) in limestone and sandstone hills throughout Thailand from 2018 to 2021. Living snails and empty shells were collected from rocks and leaf litter during the rainy seasons at Khao Bin Cave, Mueang Ratchaburi District, Ratchaburi Province; Khao Nang Panthurat Forest Park, Cha-am District, Phetchaburi Province in western Thailand; and Phu Po, Mueang Kalasin District and Phu Kum Khao, Sahatsakhan District, Kalasin Province in northeastern Thailand (Fig. 1). Most living adult specimens were drowned in water for 24 h and subsequently preserved in 70% ethanol for study of radular morphology and reproductive anatomy. Intact adult shells were used to count the number of whorls and measure shell height (**SH**), shell width (**SW**), aperture height (**AH**), aperture width (**AW**), and umbilicus width (**UW**) using digital vernier calipers. To study the genital system and radula, three adult snails from one locality per putative species were dissected under a stereomicroscope. The radulae were extracted from the buccal mass and examined under a scanning electron microscope (SEM), following the methods of Geiger et al. (2007). The material is kept in the following institutions: Natural History Museum, Mahasarakham University (Mahasarakham, Thailand; **NHMSU**); Zoological Research Collection of Muban Chombueng Rajabhat University (Ratchaburi, Thailand; **ZMCRU**); Zoological Research Collection of Chaiyaphum Rajabhat University (Chaiyaphum, Thailand; **ZCPRU**); Muséum National d’Histoire Naturelle (Paris, France; **MNHN**).

The abbreviations used are from Solem (1966) and Pholyotha et al. (2021a, 2021b): **ag**, albumen gland; **at**, atrium; **ep1**, proximal part of epiphallus (from penis side, is the parts before the insertion of the retractor muscle); **ep2**, distal part of epiphallus (is the part from the insertion of the retractor muscle to vas deferens); **fl**, flagellum; **fo**, free oviduct; **gs**, gametolytic sac (= bursa copulatrix); **hd**, hermaphroditic duct; **p**, penis; **prm**, penial retractor muscle; **pro**, prostate gland; **ut**, uterus; **v**, verge; **va**, vagina; **vd**, vas deferens.

## ﻿Results

### ﻿Systematics

**Family Camaenidae Pilsbry, 1895**

**Subfamily Bradybaeninae Pilsbry, 1934**

**Tribe Aegistini Kuroda & Habe, 1949**

#### ﻿Genus Landouria

Taxon classificationAnimaliaStylommatophoraCamaenidae

Godwin-Austen, 1918

E42D138D-4744-5372-9415-CF009807BE0E

##### Type species.

*Helixhuttonii*L. Pfeiffer, 1842 (new name for *Helixorbicula* Hutton & Benson, 1838), by original designation.

##### Type locality.

Himalaya near Simla, Mahasu, northern India (Hutton and Benson 1838).

#### ﻿ Landouria bella

Taxon classificationAnimaliaStylommatophoraCamaenidae

Nahok & S. Tumpeesuwan sp. nov.

03FA6E1C-6C54-54AA-A4E3-908EC0E7EBAD

https://zoobank.org/DDCCEAB2-071E-4EB0-83D4-077CF7EE0232

**Figure 2.**
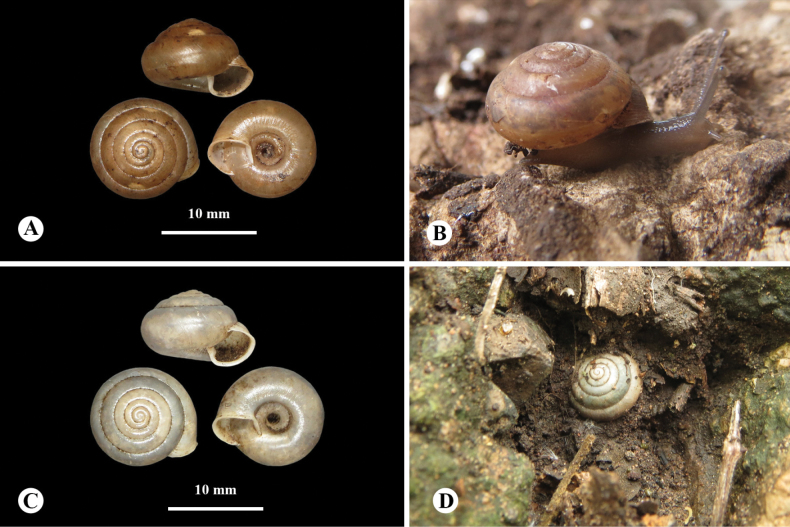
*Landouriabella* sp. nov. **A** holotype (NHMSU-00056) **B** paratype (ZCPRU-0042) **C, D** empty shell from Khao Nang Panthurat Forest Park, Phetchaburi Province (NHMSU-00058).

**Figure 3.**
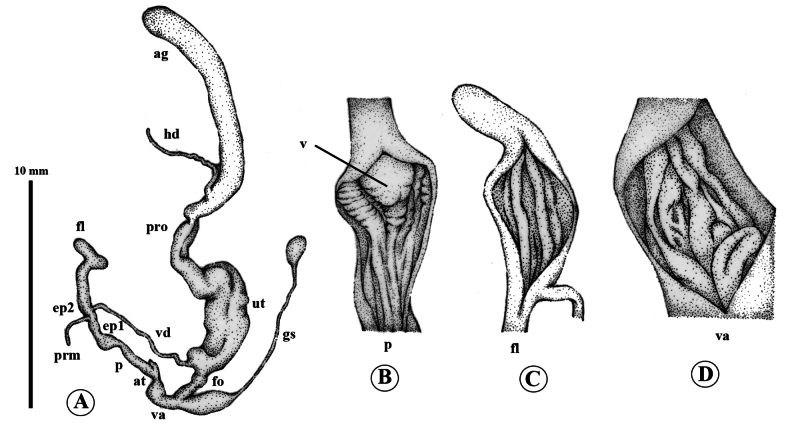
Genital anatomy of *Landouriabella* sp. nov., paratype (ZCPRU-0042) **A** whole genitalia **B–D** internal wall sculpture of **B** penis (p) **C** flagellum (fl) **D** vagina (va).

**Figure 4.**
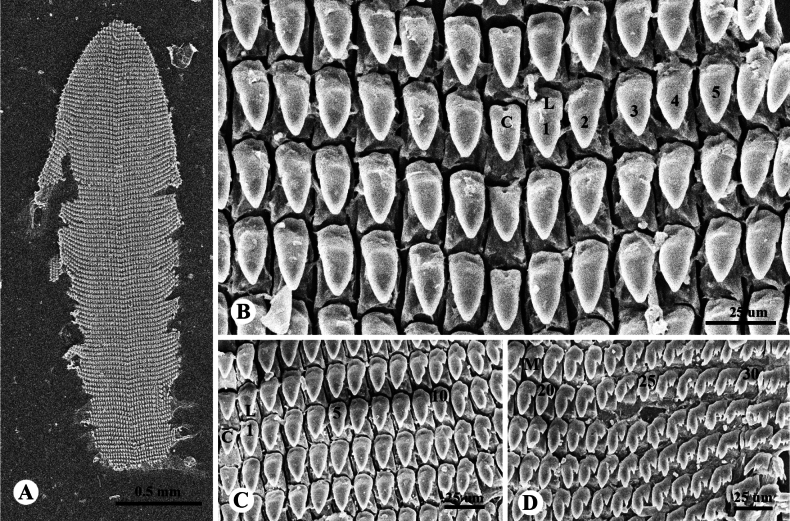
Radula morphology of *Landouriabella* sp. nov., paratype (ZCPRU-0042) **A** whole radula **B** close-up view of central tooth and lateral teeth **C** right side of central tooth and lateral teeth **D** right side of marginal teeth. Numbers indicate order of lateral and marginal teeth. Abbreviation: C = central tooth, L = lateral teeth, M = marginal teeth.

Figs 2 , 3 , 4 , Table 1

Landouria sp.16— Nahok 2020: 61; fig. 24G, I, table 3
Landouria sp.—Chanlabut and Nahok 2022: figs 2D, 3D, table 1

##### Type locality.

Thailand, Ratchaburi Province, Mueang Ratchaburi District, Khao Bin Cave, 13°35'37.56"N, 99°39'56.57"E, Isolated limestone hills, alt. 116 m. 7 July 2018. Benchawan Nahok and Utain Chanlabut leg.

##### Type specimens.

***Holotype***: • NHMSU-00056 (Fig. 2A), 1 shell, SH = 8.0 mm, SW = 11.1 mm, AH = 4.3 mm, AW = 4.3 mm, UW = 3.3 mm. ***Paratypes***: • NHMSU-00057 (Figs 2B, 3, 4) 27 shells, • ZMCRU-0001 11 shells, • ZCPRU-0041 65 shells, • ZCPRU-0042 5 living specimens preserved in ethanol, same leg. and locality as holotype, 27 June 2020. ***Other locality***: • NHMSU-00058 (Fig. 2C, D) 4 shells, • ZCPRU-0043 7 shells. Thailand, Phetchaburi Province, Cha-am District, Khao Nang Panthurat Forest Park, 12°50'20.27"N, 99°57'6.88"E, Isolated limestone hills, alt. 42 m. 13 Aug. 2017 and 7 July 2018. Benchawan Nahok and Utain Chanlabut leg.

##### Diagnosis.

Shell small, depressed-globose. Body whorl rounded; aperture slightly oval, oblique, with thick, slightly reflected lip. Thin, small lamella present on inner columellar side of aperture. Flagellum large, cylindrical, with bent tip; penis short, small, cylindrical; vagina and free oviduct short, swollen. Basal part of gametolytic sac swollen.

**Table 1.** Comparison of shells, genital systems and radulae of Thai *Landouria* species. Reference: 1 = Tumpeesuwan and Tumpeesuwan (2019); 2 = Nahok et al. (2021); 3 = this study; 4 = Schileyko (2011) (question mark = no data).

Characters	*L.strobiloides*	*L.circinata*	*L.tuberculata*	*L.trochomorphoides*	*L.chloritoides*	*L.elegans*	*L.diplogramma*	*L.bella* sp. nov.	*L.monodon* sp. nov.	*L.ptychostyla*
**Shell**:										
Shell shape	Conical-lenticular	Conical-lenticular	Slightly convex	Subconvex	Depressed	Conical	Sub-globose	Globose	Depressed- conical	Depressed-conical
Number of whorls	5 ½	6	6 ½	6	6 ½	6	5 ½	6	5 ¾	6
Peripheral band	Dark brown	Pale brown	Absent	Brown	Absent	Dark brown	Pale brown	Absent	Absent	Absent
Periphery keel	Sharp	Sharp	Moderate	Sharp	Weak	Sharp	Very weak	Absent	Slightly	Moderate
Scale sculpture	Absent	Absent	Tubercle	Scaly	Absent	Absent	Absent	Absent	Absent	Absent
Lamella at inner aperture	Absent	Absent	Absent	Absent	Absent	Absent	Absent	Present (thin)	Present (sharp)	Present (thin)
**Genital system**:										
Penis	Short, slightly stout	Long cylindrical	Swollen	Long slender	Very large and stout	Long cylindrical	Swollen	Small cylindrical	Long cylindrical	Long fusiform
Verge	?	Present	Present	Absent	Present	Absent	Present	Present	Present	Absent
Flagellum	Strobilus-shaped	Circinate-shaped	Protrusion	Slender towards tip	Narrow ovate	Water drop-shaped	Short protrusion	Cylindrical, bent tip	Cylindrical, bent tip	Shot, hook- shape
Basal part of gametolytic sac	Swollen	Thick and stout	Thin	Thin	Thick	Stout	Thick	Swollen	Swollen	Swollen
**Radula**:										
Number of rows	100	93–95	126–130	107–111	125–129	110–114	121–125	103–107	136–140	?
Number of teeth in each row	3–53	55–63	79–87	65–73	75–83	69–77	61–69	63–71	63–71	?
- Lateral teeth	8–10	8–10	11–13	8–10	11–13	12–14	7–9	6–8	12–14	?
- Marginal teeth	15–17	19–21	28–30	24–26	26–28	22–24	23–25	22–24	19–21	?
Central tooth	Elongate-lanceolate	Tongue-shaped	Slender-lanceolate	Short-lanceolate	Lanceolate	Tongue-shaped	Triangular	Lanceolat	Triangular	?
References	1	2	2	2	2	2	2	3	3	4

##### Description.

(empty shells = 115, living specimens = 5). ***Shell*** (Fig. 2) dextral, small, depressed-globose; body whorl large, well rounded. Whorls six; suture deep; apex obtuse; spire much elevated. Protoconch smooth; teleoconch with fine, irregular wrinkles, without scaly processes, last whorl with indistinct, incised spiral lines; umbilical side of last whorl with spiral lines more distinct. Umbilicus deep, very wide. Shell dimensions of specimens from type locality (*N* = 104; mean values in parentheses): shell height 5.7–9.4 mm (6.69 ± 0.71 mm), shell width 8.5–12.2 mm (10.54 ± 0.65 mm), aperture height 3.2–5.3 mm (4.47 ± 0.38 mm), aperture width 2.9–5.1 mm (4.08 ± 0.34 mm), and umbilicus width 2.7–5.4 mm (3.60 ± 0.33 mm). Shell dimensions of specimens from Khao Nang Panthurat (*N* = 11): shell height 5.81–7.09 mm (6.45 ± 0.64 mm), shell width 9.59–10.87 mm (10.23 ± 0.64 mm), aperture height 4.13–4.73 mm (4.43 ± 0.30 mm), aperture width 3.64–4.38 mm (4.01 ± 0.37 mm), and umbilicus width 3.25–3.83 mm (3.54 ± 0.29 mm).

***Genital system*** (*N* = 3) (Fig. 3). Atrium short. Penis small, cylindrical, shorter than flagellum, internally with four smooth, longitudinal pilasters, which gradually become corrugated at distal part close to verge. Verge short, swollen (Fig. 3B). Penial retractor muscle present. Proximal part of epiphallus (ep1) approximately equal to length of penis and vagina combined, but thicker and larger than penis. Distal part of epiphallus (ep2) extremely short. Flagellum apically joins epiphallus, more than twice as long as penis, regularly long-cylindrical, bent at distal end, internally with three smooth, longitudinal pilasters (Fig. 3C). Vas deferens a thin, cylindrical tube, laterally entering epiphallus. Vagina short, distally globularly dilated, internally with five thick, longitudinal pilasters (Fig. 3D). Free oviduct shorter than vagina. Proximal part of gametolytic sac very stout, swollen, with a long, narrow, cylindrical tube, and with at its distal end sac-like, small, swollen, and spherical. Prostate gland very long; uterus long, swollen.

***Radula*** (*N* = 3) Comprises 103–107 transverse rows with 63–71 teeth per row; radular formula: (22–24)+(6–8)+1+(6–8)+(22–24). Central tooth usually symmetric, unicuspid, lanceolate (Fig. 4B). Lateral teeth quite like central tooth, but oblique, larger, and longer. Teeth on both sides begin to transform into indistinct bicuspid marginal teeth with tiny ectocone at tooth number 6–8 (Fig. 4C) and gradually change from bicuspid to tricuspid; endocone small; mesocone large, lanceolate; ectocone triangular, with 2–4 tiny cusps (Fig. 4D).

##### Etymology.

Specific epithet “*bella*” is derived from the Latin word “bellus”, which means lovely fine, pretty, and beauty. This name refers to the lovely shell of the new species.

##### Habitat.

This species inhabits isolated limestone hills, where it lives on rocks and the ground in a natural forest at the foot of limestone cliffs.

##### Distribution.

*Landouriabella* sp. nov. is currently known only from Khao Bin Cave, Ratchaburi Province, and Khao Nang Panthurat Forest Park, Phetchaburi Province, in western Thailand (Fig. 1).

##### Remarks.

The new species is distinguished from other species of *Landouria* in Thailand by its depressed-globose shell, the presence of a thin, small columellar lamella on the inner side of the aperture, and the absence of an angular and peripheral keel (Fig. 2). Its genital system is distinguished by its flagellum, which is regularly cylindrical, with a bent tip. The internal structure of the penis has a rounded swollen verge and resembles that of *L.circinata*, as well as *L.tuberculata* from Loei, *L.chloritoides* from Khon Kaen, and *L.diplogramma* from Nakhon Ratchasima, northeastern Thailand, but the new species differs in having the proximal part of the penis with thin longitudinal pilasters and the distal part which becomes gradually corrugated (Fig. 3B). Most radular characteristics are quite similar to those of other species, except for the central tooth, which is lanceolate (Fig. 4B).

#### ﻿ Landouria monodon

Taxon classificationAnimaliaStylommatophoraCamaenidae

Nahok & C. Tumpeesuwan sp. nov.

DE4F9005-B56C-5A65-8A52-F8040F91FB8D

https://zoobank.org/11969825-6F9C-4FD2-BB73-5936301502E8

**Figure 5.**
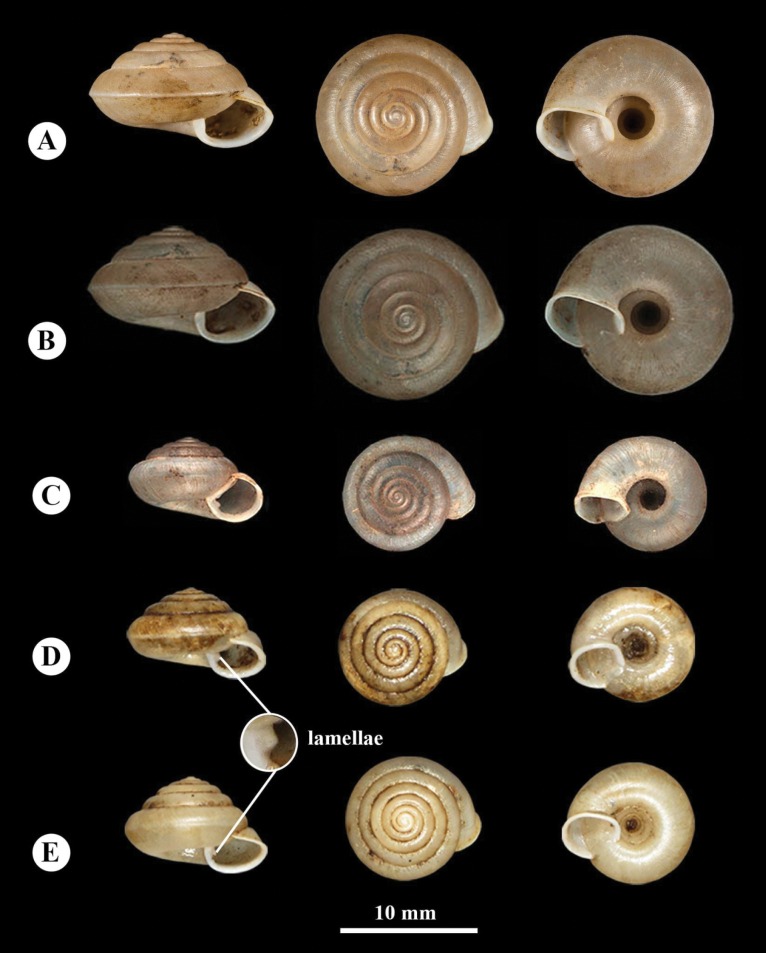
**A, B***Helixgoniochila*L. Pfeiffer, 1862 syntype (MNHN-2000-1901) **C***Helixptychostyla*L. Pfeiffer, 1862 syntype (MNHN-2000-1974) **D, E***Landouriamonodon* sp. nov. **D** holotype (NHMSU-00059) **E** empty shell from Phu Kum Khao, Kalasin Province (NHMSU-00062). Photographs by: **A** Manuel Caballer (MNHN) E-Recolnat Project (ANR-11-INBS-0004); **C** Brabant D (MNHN); **D–E** Benchawan Nahok.

**Figure 6.**
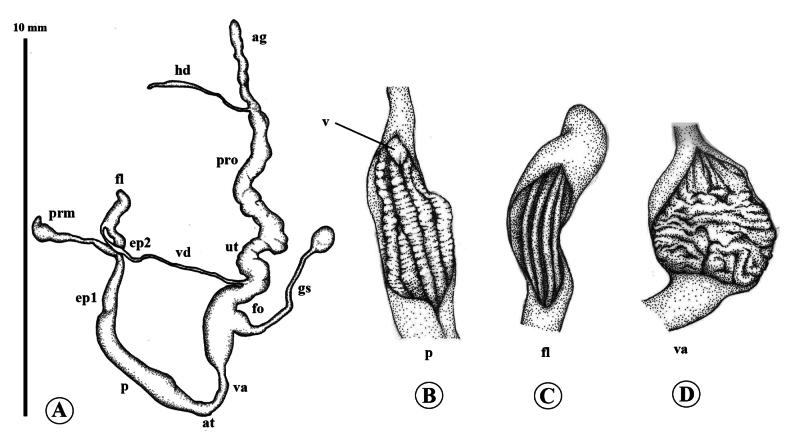
Genital anatomy of *Landouriamonodon* sp. nov., paratype (NHMSU-00061) **A** whole genitalia **B–D** internal wall sculpture of **B**, penis (p) **C** flagellum (fl) **D** vagina (va).

**Figure 7.**
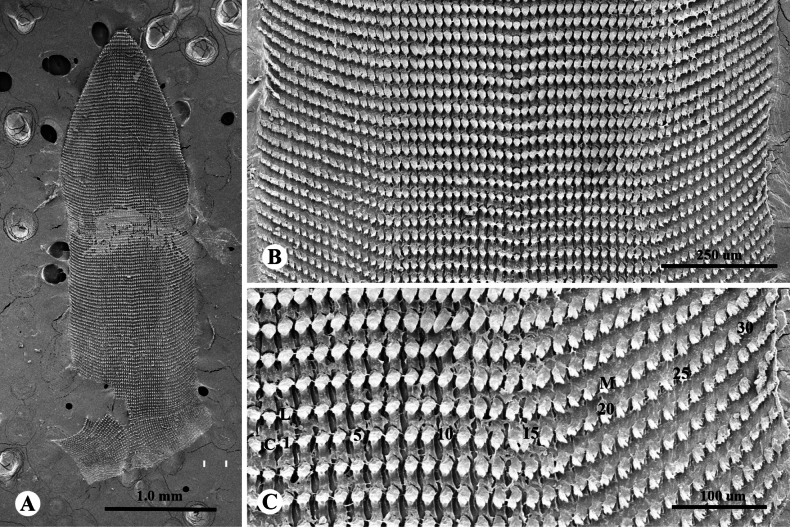
Radula morphology of *Landouriamonodon* sp. nov., paratype (NHMSU-00061) **A** whole radula **B** view of transverse rows of radula **C** right side of central tooth, lateral teeth, and marginal teeth. Numbers indicate order of lateral and marginal teeth. Abbreviation: C = central tooth, L = lateral teeth, M = marginal teeth.

Figs 5D–E , 6 , 7 , Tables 1 , 2

Landouria sp. 1—Ounchareon 2016: 27–32, fig. 4.1A, B, table 4.3
Landouria sp. 2—Nahok 2020: 43–44, fig. 23B, C, table 3

##### Type locality.

Thailand, Kalasin Province, Mueang Kalasin District, Phu Po, 16°37'10.42"N, 103°37'55.59"E, Sandstone hills, alt. 241 m. 17 Jun. 2017. Benchawan Nahok and Utain Chanlabut leg.

##### Type material.

***Holotype***: • NHMSU-00059 (Fig. 5D). 1 shell, SH = 6.4 mm, SW = 10.3 mm, AH = 3.4 mm, AW = 4.0 mm, UW = 2.9 mm. ***Paratypes***: • NHMSU-00060, (Figs 6, 7) 12 shells, • ZCPRU-0045 7 shells, • NHMSU-000613 living specimens preserved in ethanol, • ZCPRU-0046 2 living specimens preserved in ethanol, same leg. and locality as holotype, 26 Sep. 2017.

##### Other material.

• NHMSU-00062 (Fig. 5E) 4 shells, • ZCPRU-0047 4 shells. Thailand, Kalasin Province, Sahatsakhan District, Phu Kum Khao, 16°41'41.98"N, 103°31'34.43"E, Sandstone hills, alt. 267 m, 17 June 2017. Benchawan Nahok and Utain Chanlabut leg.

##### Diagnosis.

Shell small, depressed-conical, slightly keeled; aperture oval, oblique, with thick, reflected lip. Thick prominent lamella present on inner columellar side of aperture. Flagellum short, bend at apical portion. Penis long, large, cylindrical. Free oviduct short; vagina long, distally swollen. Basal part of gametolytic sac enlarged, swollen.

##### Description.

(empty shells = 28, living specimens = 5). ***Shell*** (Fig. 5D, E) dextral, small, depressed-conical. Whorls 5¾–6¼; apex obtuse. Body whorl slightly keeled. Protoconch almost smooth; teleoconch with irregular wrinkles, indistinct incised spiral lines, without scaly processes. Prominent but tiny lamella present on inner columellar side of aperture. Umbilicus deep, wide. Shell dimensions of specimens from type locality (*N* = 20; mean values in parentheses): shell height 3.10–6.60 mm (4.68 ± 1.04 mm), shell width 5.10–10.40 mm (7.67 ± 1.24 mm), aperture height 2.10–3.50 mm (2.64 ± 0.39 mm), aperture width 2.20–4.10 mm (3.26 ± 0.55 mm), and umbilicus width 2.20–3.00 mm (2.49 ± 0.20 mm). Shell dimensions of specimens from Phu Kum Khao (*N* = 8): shell height 3.64–4.68 mm (4.85 ± 0.88 mm), shell width 6.43–7.67 mm (7.75 ± 1.33 mm), aperture height 2.25–2.64 mm (2.80 ± 0.35 mm), aperture width 2.71–3.26 mm (3.15 ± 0.48 mm), and umbilicus width 2.29–2.49 mm (2.60 ± 0.20 mm).

***Genital system*** (*N* = 3) (Fig. 6). Atrium short. Penis cylindrical, longer than flagellum, internally with four corrugated, longitudinal pilasters, distally giving rise to densely interlocked longitudinal rows to verge. Verge small, short (Fig. 6B). Penial retractor muscle present. Proximal part of epiphallus (ep1) shorter than half of penis length. Distal part of epiphallus (ep2) very short. Flagellum approximately as long as proximal part of epiphallus, regularly short-cylindrical, slightly bent at the tip, internally with four smooth, longitudinal pilasters (Fig. 6C). Vas deferens a thin cylindrical tube, apically entering epiphallus. Vagina long, distally swollen, internally with thick, undulating, transverse pilasters (Fig. 6D). Free oviduct shorter than vagina. Gametolytic sac swollen at base, short cylindrical tube, and with swollen spherical sac at distal end. Prostate gland long; uterus swollen.

***Radula*** (*N* = 3). Comprises 136–140 transverse rows with 63–71 teeth per row (Fig. 7B); radular formula: (19–21)+(12–14)+1+(12–14)+(19–21). Central tooth usually symmetric, small, tricuspid, triangular. Lateral teeth short, bicuspid, with tiny ectocone and gradually changing into tricuspid marginal teeth; endocone small; mesocone large, long, with curved margins; ectocone triangular, with 2–4 tiny cusps (Fig. 7C).

##### Etymology.

Specific epithet “*monodon*” is derived from the Greek words, “monos”, one or single, and “odous”, tooth, and refers to the single prominent lamella on the inner columellar side of aperture.

##### Habitat.

This new species lives on sandstone hills under leaf litter, in rock crevices, hollow trees, etc. at bases of hills in dry, dipterocarp forest.

##### Distribution.

*Landouriamonodon* sp. nov. is currently known from separate sandstone hills, at Phu Po and Phu Kum Khao, Kalasin Province, northeastern, Thailand (Fig. 1).

##### Remarks.

*Landouriamonodon* sp. nov. differs from other Thai species of *Landouria* by its tiny but prominent columellar lamella on the inner side of the aperture (Fig. 5D, E). Its genital system and radula are quite similar to *L.diplogramma* from Nakhon Ratchasima, northeastern Thailand, but the new species differs in having its penis not divided into two short portions and its vagina thicker (Fig. 6). The radular central tooth is triangular but differs by its smaller size (Fig. 7). Conchologically, *L.monodon* sp. nov. closely resembles *Helixptychostyla*L. Pfeiffer, 1862, whose types specimen are in MNHN (MNHN-IM-2000-1974; Fig. 5C). (https://www.gbif.org/occurrence/1019688902). Still, the two species differ by many characters: (1) parietal callus prominent in *H.ptychostyla*, but faint or absent in *L.monodon* sp. nov.; (2) last whorl and lip descending in front in *L.monodon* sp. nov.; (3) growth lines prominent in *H.ptychostyla*, but faint in *L.monodon* sp. nov.; (4) umbilical keel absent in *H.ptychostyla*, but prominent in *L.monodon* sp. nov.

Moreover, the type material of *H.ptychostyla*L. Pfeiffer, 1862 likely comes from the Henri Mouhot expedition to Thailand and Laos, which included Bangkok, Wat Phra Phutthachai Saraburi, Korat, Chaiapume, Leute, Kenne Tao, Paklaie, and Thadua (see Inkhavilay et al. 2019: fig. 1), which is over 150 km away from the type locality of *L.monodon* sp. nov.

Recently, Schileyko (2011) proposed that *Helixptychostyla* was preoccupied by von Martens (1860) and indicated that *H.goniochila*L. Pfeiffer, 1862 (Figs 5A, B) is a synonym of *H.ptychostyla* von Martens, 1860. He used *Thaitropisptychostyla* (von Martens, 1860) as the available name for this taxon, whereas *T.ptychostyloides* was proposed as *nomen novum* for *Helixptychostyla*L. Pfeiffer, 1862 (Table 2).

**Table 2.** History of Scientific name changes of *Landouriaptychostyla* (von Martens, 1860) and *L.ptychostyloides* (Schileyko, 2011).

Authors	*L.ptychostyla* (von Martens, 1860)	*L.ptychostyloides* (Schileyko, 2011)
von Martens (1860)	*Helixptychostyla* von Martens, 1860	—
Pfeiffer (1862)	*Helixgoniochila* Pfeiffer, 1862	*Helixptychostyla* Pfeiffer, 1862
Panha (1996)	*Aegistagoniochila* (L. Pfeiffer, 1862)	—
Schileyko (2004)	*Thaitropisgoniochila* (L. Pfeiffer, 1862)	—
Schileyko (2011)	*Thaitropisptychostyla* (von Martens, 1860)	*Thaitropisptychostyloides*, Schileyko, 2011
Nurinsiyah et al. (2019)	*Landouriaptychostyla* (von Martens, 1860)	*Landouriaptychostyloides* (Schileyko, 2011)

## ﻿Discussion

A unique characteristic shared by all members of *Landouria* in Thailand is the absence of a dart sac and stimulatory organs, a character combination that is thought to define the family Camaenidae (Páll-Gergely et al. 2013). We agree with Köhler et al. (2019), Nurinsiyah et al. (2019), Tumpeesuwan and Tumpeesuwan (2019), and Páll-Gergely et al. (2020) in rejecting the proposal by Hirano et al. (2014) to treat *Landouria* as a junior synonym of *Aegista*, on account that *Aegista* possesses both a dart sac and stimulatory organs and its geographic distribution is disjunct from *Landouria*. *Landouria* and *Aegista* are two well-differentiated genera, with *Landouria* being distinguished by, for example, its small, depressed shell; broad, open umbilicus; and the presence of a flagellum of various shapes.

*Landouriabella* sp. nov. differs from all other recently verified Thai *Landouria* species by its depressed-globose shell. Other Thai *Landouria* species usually have a slight to pronounced angular keel at the periphery, as seen in *L.monodon* sp. nov. The existence of a small, thin columellar lamella on the inner side of the aperture of *L.bella* sp. nov. (Fig. 2A, C) resembles *L.monodon* sp. nov. (Fig. 5D, E), but this columellar lamella is prominent in the latter species.

We present below an updated and improved dichotomous identification key to the *Landouria* species of northeastern Thailand based on Nahok et al. (2021).

### ﻿Key to Thai *Landouria* species by shell morphology

**Table:** 

1	Shell without peripheral keel	**2**
–	Shell with peripheral keel	**3**
2	Shell depressed-globose, without brown band	***L.bella* sp. nov.**
–	Shell low-conical, with brown band	***L.diplogramma***
3	Inner side of aperture with prominent columellar lamella	***L.monodon* sp. nov.**
–	Inner side of aperture without columellar lamella	**4**
4	Peripheral keel blunt	***L.chloritoides***
–	Peripheral keel sharp	**5**
5	Peripheral keel slightly sharp, shell surface with numerous tiny tubercles	***L.tuberculata***
–	Peripheral keel very sharp, shell surface without tubercles	**6**
6	Shell with low spire, shell surface with radial scaly processes	***L.trochomorphoides***
–	Shell with high spire, shell surface without radial scaly processes	**7**
7	Keel with downward bent rim	***L.elegans***
–	Keel without downward bent rim	**8**
8	Suture indented, growth line obvious	***L.strobiloides***
–	Suture not indented, growth line obscure	***L.circinata***

### ﻿Key to Thai *Landouria* species by genital characters

**Table:** 

1	Penis shorter than vagina; flagellum strobilus-like	***L.strobiloides***
–	Penis longer than vagina; flagellum non-strobilus-like	**2**
2	Flagellum circinate	***L.circinata***
–	Flagellum a short protrusion: ovate, slender, or long cylindrical	**3**
3	Inner sculpture of penis with parallel, transverse folds	***L.trochomorphoides***
–	Inner sculpture of penis with longitudinal pilasters	**4**
4	Rounded verge absent	***L.elegans***
–	Rounded verge present	**5**
5	Epihallus clearly divided into two portions (ep1 and ep2)	***L.diplogramma***
–	Epihallus not clearly divided into two portions (ep2 is very short)	**6**
6	Vagina as long as free oviduct	***L.chloritoides***
–	Vagina length not as long as free oviduct	**7**
7	Basal part of gametolytic sac slightly swollen	***L.tuberculata***
–	Basal part of gametolytic sac swollen	**8**
8	Inner sculpture of vagina thick longitudinal folds pilasters	***L.bella* sp. nov.**
–	Inner sculpture of vagina thick undulating transverse pilasters	***L.monodon* sp. nov.**

## Supplementary Material

XML Treatment for Landouria

XML Treatment for Landouria bella

XML Treatment for Landouria monodon

## ﻿Additional information

### Author ORCIDs

Benchawan Nahok ￼ https://orcid.org/0009-0009-1788-6788

Utain Chanlabut ￼ https://orcid.org/0000-0001-8275-7434

Sakboworn Tumpeesuwan ￼ https://orcid.org/0000-0001-5900-7862

Chanidaporn Tumpeesuwan ￼ https://orcid.org/0000-0003-2066-1271

### Data availability

All of the data that support the findings of this study are available in the main text.
